# Survival, mortality, and related comorbidities among COVID-19 patients in Saudi Arabia: A hospital-based retrospective cohort study

**DOI:** 10.15537/smj.2022.43.8.20220182

**Published:** 2022-08

**Authors:** Mohammad A. AL-Ghamdi, Rajaa M. Al-Raddadi, Iman K. Ramadan, Ahmad A. Mirza, Hanan A. Alsaab, Hani F. Alobaidi, Mohammed Y. Bin Hayd

**Affiliations:** *From the Department of Preventive Medicine (AL-Ghamdi), Health Surveillance Center, Ministry of Health, Al Medina Al Munawarah; from the Department of Community Medicine (Al-Raddadi, Ramadan, Bin Hayd), Faculty of Medicine; from the Department of Otolaryngology–Head and Neck Surgery (Mirza), Faculty of Medicine in Rabigh, King Abdulaziz University; from the Department of Medical Records (Alsaab), King Abdullah Medical Complex, Ministry of Health, Jeddah; from the Department of Preventive Dentistry (Alobaidi), Ministry of Health, Taif, Kingdom of Saudi Arabia; from the Department of Community Medicine (Ramadan), Faculty of Medicine, Al-Azhar University, Cairo, Egypt; and from the Department of Otolaryngology–Head and Neck Surgery (Mirza), Temerty Faculty of Medicine, University of Toronto, Toronto, Canada.*

**Keywords:** COVID-19, SARS-CoV-2, mortality, inpatients, Saudi Arabia

## Abstract

**Objectives:**

To assess the survival of COVID-19 patients in Saudi Arabia and to investigate possible mortality predictors.

**Methods:**

This is a retrospective cohort study involving 248 patients with severe acute respiratory syndrome coronavirus-2 who were admitted to the primary COVID-19 referral hospital in Jeddah between March and June of 2020. Socio-demographic characteristics, comorbidities, laboratory investigations, management protocols, complications, treatment options, and mortality data were extracted from electronic medical records. The time analysis began at the first signs of illness thorough discharge or death.

**Results:**

Our study showed that in-hospital complications including heart failure followed by acute renal failure had the largest effect size on mortality (*p*<0.001). Elderly patients and those with comorbid asthma had a higher risk of death. Non-survivors presented more commonly with shortness of breath and fever than survivors. High D-Dimer level was a marginally significant indicator of mortality in the studied population (*p*=0.05). We did not find a significant benefit in relation to any treatment option.

**Conclusion:**

Age, asthma, some in-hospital complications are important survival indicators in hospitalized COVID-19 patients. The controllable co-factors should be monitored and managed by healthcare workers to reduce mortality rates in those hospitalized with COVID-19.

**S**evere acute respiratory syndrome coronavirus 2 (SARS-CoV-2), which causes coronavirus disease 2019 (COVID-19), accounted for more than 4.1 million with 290,000 fatalities globally as of May 11th, 2020.^1^ World Health Organization (WHO) has received reports of 781,168 confirmed cases of COVID-19 from Saudi Arabia with 9,179 fatalities as of June 17th, 2022.^2^ This outbreak became a public health crisis requiring international public health and government involvement.

**C**oronavirus disease-19 vary in its clinical presentation from no symptoms to severe manifestations requiring hospitalization. Coronavirus disease-19 clinical manifestations are mostly defined by a cluster of flu-like symptom (fever, cough, dyspnea, myalgia, exhaustion, diarrhea, and smell/taste problems). Acute coronary disease, acute respiratory distress syndrome, acute kidney injury, and one or multiple organ failures or malfunction are all common life-threatening complications of the condition.^3^ These serious consequences appear to be exacerbated in COVID-19 individuals who are older (>60 years old) or have one or more comorbidities.^4^ According to clinical research data, 58% of patients with COVID-19 has underlying disorders such as hypertension, diabetes, cardiovascular disease (CVD), and chronic obstructive pulmonary disease.^4^ Two recent meta-analyses combined COVID-19 research data from different countries elucidated that some demographic characteristics (such as male gender and older age), diabetes mellitus, hypertension, CVD, and malignancy are linked to unfavorable outcomes.^5,6^ The case fatality ratio varies across nations; currently, it ranges from 5.6% in Mexico to <0.1%.^7^

Some retrospective studies carried out in Saudi Arabia examined the demographic and clinical characteristics of COVID-19 patients and provided comparable results.^8,9^ Approximately, 64% of COVID-19 non-survived patients had multiple comorbidities.^8^ Approximately 72% of COVID-19 patients required hospitalization, and almost 5% received intensive care. The mortality rate was estimated to be roughly 0.7%.^9^ However, this seems to be underestimated as clinical outcomes including mortality could not fully assessed due to the limited study period. Thus, a study with a longer study period on a large-scale in the region is needed to provide insight into mortality rate and predictions of death.

In Saudi Arabia, studies describing the clinical characteristics of COVID-19 patients are limited by small sample size.^10,11^ Additionally, regional data on the predictors and risk factors of COVID-19 disease severity and mortality have been insufficient and limited with a short study period.^9^ Elucidating risk factors support disease detection and case management and ultimately improve the survival. King Abdullah Medical Complex was the main referral center to treat COVID-19 patients in the city of Jeddah where a wide range of populations (with different ethnicities and nationalities) lives. This paper examined comorbid conditions, laboratory investigations, course of the disease, management protocols, and treatment options in relation to mortality rates in patients afflicted with the wide spreading COVID-19 disease who were admitted to the primary referral center in Jeddah.

## Methods

This hospital-based descriptive retrospective cohort study examined clinical characteristic and outcomes of COVID-19 cases admitted to King Abdullah Medical Complex Center, Jeddah, Saudi Arabia between March and June 2020. Patients aged 18-years and older with reverse-transcriptase polymerase chain reaction (RT-PCR)-confirmed COVID-19 admitted to the center were included in the study. Patients with unavailable or incomplete data (>5% of missing variables of interest) were excluded. Ethical approval was obtained from the Ethics Committee in the Ministry of Health (MOH), Jeddah, Saudi Arabia. All patients’ information in this study was anonymized for patient confidentiality. The study was carried out in accordance with the Declaration of Helsinki principles.

Demographic data, comorbid conditions, laboratory investigations, disease course, management protocols, and treatment options were extracted from electronic medical records. The onset of disease was defined as the time when the first symptom manifested. Clinical outcomes were followed up to discharge from hospital or death.

### Statistical analysis

Using STATA software version 13.1 (StataCorp. 2013. Stata Statistical Software: Release 13. College Station, TX: StataCorp LP), a sample size was calculated. The sample size was based on the proportion of diabetes comorbidity in 2 hospitalized COVID-19 groups: severe COVID-19 group (0.19) and non-severe COVID-19 group (0.11).^12^ Considering a power of 80% and α-error of 0.05, the calculated sample size was 140 participants. The sample size was then increased to 250 to compensate for those excluded charts that were unavailable or incomplete (>5% of missing variables of interest). Sample sizes calculated according to the proportions of hypertension and CVD with severe COVID-19 were smaller than 250 participants.^12,13^ Furthermore, on the basis of a previous investigation that assessed the proportion of hypertension and other cardiovascular comorbidities among deceased COVID-19 patients against recovered patients, the estimated sample size was smaller than the target sample size.^14^ In addition, we used log-rank test comparing 2 survival rates with alpha error of 0.05, power of 0.8, and hazard ratios that were based on multiple studies which explored the effect of multiple comorbidities on mortality.^15-17^ This yielded a sample size of less than 250.

Using a systematic random sampling approach, the population size was divided by the desired sample size. A random patient’s chart was selected based on a random number generator after which every fourth file was selected.

A standardized data collection form from the WHO/International Severe Acute Respiratory and Emerging Infection Consortium case record form for COVID-19 was used a data source. The form was composed of 3 modules: i) presentation/admission case report form, ii) daily case report form, and iii) outcome case report form.

Data were exported from an Excel sheet into the STATA software, version 13 for analysis. Descriptive data was presented by mean and standard deviation for normally distributed continuous variables, and frequencies and percentages for categorical variables. The normal level of each laboratory investigations was defined: hemoglobin for males 14.0-18.0 g/dL and for females 12.0-16.0 g/dL, white blood counts (WBCs) 3.5-12.0x109/L, platelets (x10^^^9/L) 150-400, activate partial thromboplastin time (aPTT) 25-40 sec, partial thromboplastin time (PT) 0.9-1.1 sec, international normalized ratio (INR) 0.9-1.1, glucose 3.9-6.1 mmol/L, D-Dimer <0.5 mg/L, ferritin 20-200 ng/mL, hemoglobin A1C 4.0-6.4%.

Bivariate analysis for the association between the dependent variable (time-to-death) and other independent variables was carried out using log-rank test to determine the difference between proportions for normally distributed variables. Kaplan–Meir survival curves were plotted to graph the bivariate association between the dependent variable (time-to-death) and explanatory variables. A Cox proportional hazard regression model was constructed to identify the independent predictors of time-to-death among the study participants. We used a stepwise technique and included all variables which showed statistical significance with time-to-death in bivariate analysis. We used an entry *p*-value of 0.10 and a removal *p-*value of 0.101 for variable selection. Statistical significance was determined at *p*-value of <0.05 with the 2-tail probability.

## Results

Of 248 participants enrolled in study, most were male (76%), most were non-Saudi (63%), and most were of Arab origin. Approximately half were more than 50 years of age and 12 were healthcare workers. Nearly one-third (35%) had history of diabetes (either type I or type II), 30% had hypertension, and 10% had chronic cardiac disease. In total, 34% of the participants used diabetic medications (oral hypoglycemics or insulin), nearly one-third (29%) used medications for hypertension, and fewer than 5% used steroids, antibiotics, or anticoagulants before admission. The most prevalent symptoms at presentation were fever (68%) and shortness of breath (54%). Table 1 demonstrates baseline vital signs of the study subjects.

**Table 1:** - Socio-demographic characteristics, chronic medical illnesses, home medications, presenting coronavirus disease symptoms, and baseline vital signs of the study subjects (N=248).

Variable	n	%
Age,mean ±SD (range)^*^	49.38 ±15.46 (18 – 93)	
***Gender***		
Female	60	24.2
Male	188	75.8
***Nationality***		
Non-Saudi	155	62.5
Saudi	93	37.5
***Ethnicity***		
Arab	138	55.7
East Asian and Southeast Asian	17	6.9
South Asian	73	29.4
Black	16	6.5
White	4	1.6
***Employment status***		
Hhealthcare worker	12	4.8
Others	236	95.2
Body mass index, mean ±SD (range)^*^	29.2 ±6.42 (18.4 –52.0)	
***Obesity***		
No	221	89.1
Yes	27	10.9
***Home medications***		
Anti-hypertensive drugs	72	29.0
Antibiotics “before admission”	10	4.0
Anticoagulants	6	2.4
Steroids^*^	4	1.6
Hypoglycemic drugs (oral hypoglycemics)	42	16.9
Hypoglycemic drugs (insulin)	43	17.3
***Chronic medical illnesses***		
Chronic cardiac disease	24	9.7
Hypertension	74	29.8
Diabetes mellitus type I	17	6.9
Diabetes mellitus type II	69	27.8
Asthma	6	2.4
Renal diseases	9	3.6
Hepatic diseases	2	0.8
Neurological disorders	5	2.0
Malignancy	1	0.4
***Presenting COVID-19 symptoms***		
Fever	168	67.7
Cough	52	31.1
Sore throat	30	12.1
Shortness of breath	135	54.4
Chest pain	16	6.5
Fatigue	9	3.6
Muscle ache	13	5.2
Runny nose	13	5.2
Wheeze	2	0.8
Low chest wall indrawing	2	0.8
Headache	25	10.1
Altered consciousness	4	1.6
Loss of smell sensation	11	4.4
Loss of taste sensation	12	4.8
***Baseline vital signs***	mean ±SD (range)	
Temperature (Celsius)^*^	37.32 ± 0.75 (36-39.4)	
Respiratory rate^*^	20.65 ±4.31(9-40)	
Heart rate^*^	92.71 ±16.50 (39-153)	
Systolic blood pressure^*^	128.98 ±16.87 (90-191)	
Diastolic blood pressure^*^	73.99 ±10.81(41-115)	
Oxygen saturation (%)^*^	95.02 ± 6.91(20-100)	

^*^ The variable has missing data. SD: standard deviation

Mean (SD) hemoglobin level was 12.8 (2.3) g/dL, with three-quarters (75%) of the participants had normal hemoglobin levels. Most patients (95%) had leukocytosis. More than two-thirds (72%) of the group presented with normal platelet levels, yielding a group mean (SD) platelet count of 245.7 (94.8) x10^^^9/L. Levels of coagulation factors (aPTT and PT) were largely normal; however, the majority of study subjects had high levels of INR (56%) and D-Dimer (79%), with mean (SD) of 1.1 (0.2) for INR and 3.8 (10.5) for D-Dimer parameter. Kidney and liver function tests were also largely normal at baseline. Finally, only one-third (34%) of the participants presented with normal glucose levels, whereas over twice as many (83%) presented with elevated levels of hemoglobin A1C.

This study explored in-hospital management protocols and medications used during the course of illness. A total of 89 (36%) participants required intensive care unit (ICU) admission. Few required non-invasive (47;19%) or invasive ventilation (36; 15%), tracheostomy (15; 6%), or renal replacement therapy (7; 3%). The most common hospital-administered medications were broad-spectrum antibiotics (60%) and heparin (44%). Others were chloroquine (33%) and antiviral agents including Ribavirin (10%) and Lopinavir-Ritonavir (23%).

**Figure 1:**
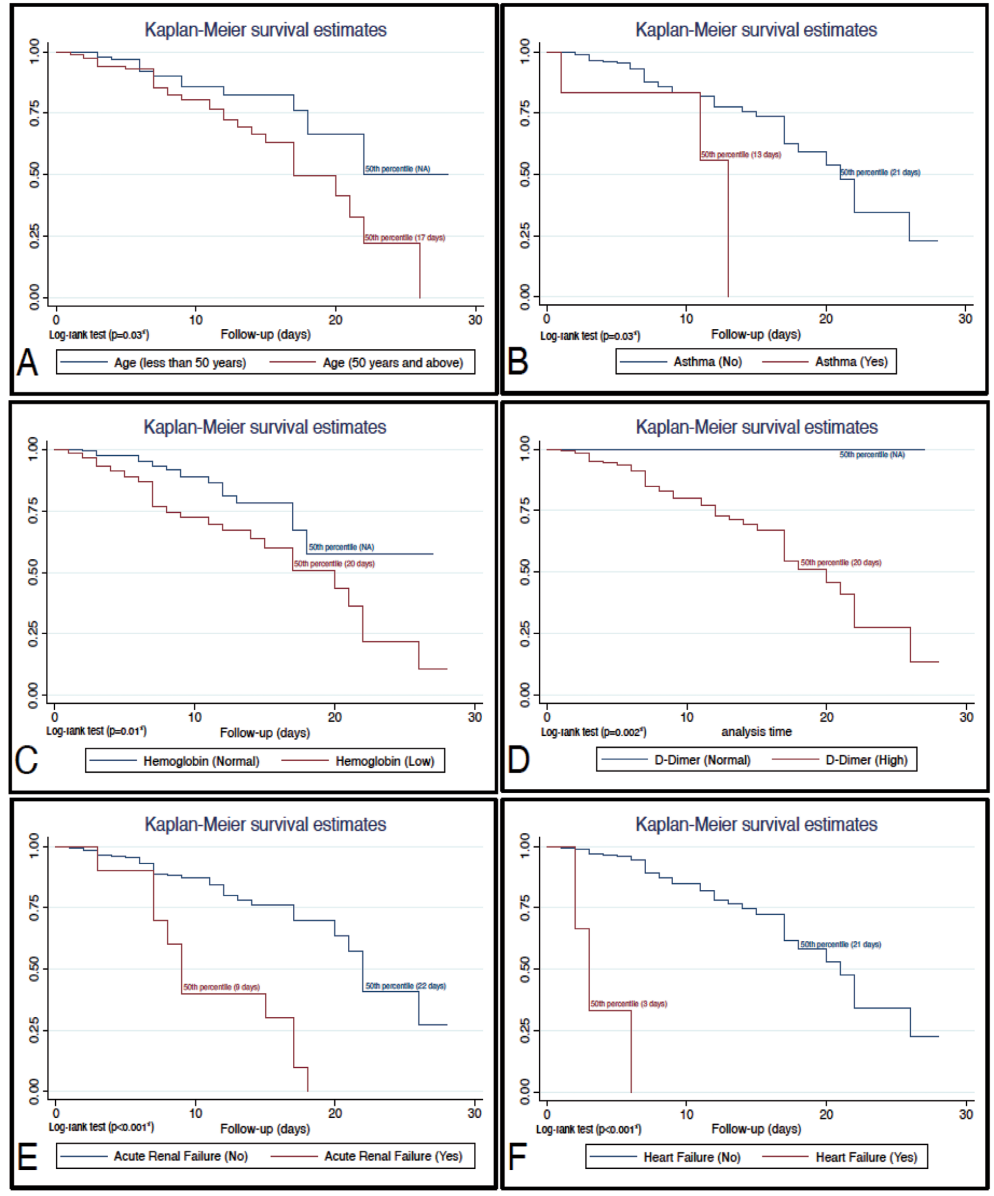
- Kaplan-Meier survival curve of significant predictors of mortality: **A**) age wise stratification; **B**) presence of comorbid asthma, **C**) normal versus low hemoglobin level; **D**) D-dimer level-based stratification; **E**) presence of acute renal failure as a complication; **F**) presence of heart failure as a complication. ^*^Statistically significant at *p*<0.05. NA: not applicable, because of lack of event in the specified cohort at the 50th percentile.

Kaplan-Meier survival curves show that median survival was 21 days over a 30-day follow-up period. Survival was worse among those aged more than 50 years compared to the remainder of the participants. The older group experienced more events and showed shorter survival times (*p*=0.03; Figure 1A). Table 2 shows how comorbid chronic diseases and COVID-19 symptoms were related with survival outcome. Chronic cardiac disease (*p*=0.002) and (*p*=0.03) asthma were significantly related to mortality. The presence of fever (*p*=0.03) and shortness of breath (*p*=0.007) were significantly related to mortality.

**Table 2:** - Distribution of survival status in relation to the demographics, chronic medical illnesses, and COVID-19 symptoms in the study population (N=248).

Parameter	Survivals		Non-survivors		Long rank test	*P*-value
	n	%	n	%		
Age						
<50 years	111	89.5	13	10.5	5.0	0.03^*^
50+ years	91	74.0	32	26.0		
Gender						
Female	48	80.0	12	20.0	0.24	0.62
Male	155	82.5	33	17.6		
***Chronic medical illnesses***						
Chronic cardiac disease						
No	190	84.8	34	15.2	10.05	0.002^*^
Yes	13	54.2	11	45.8		
Hypertension						
No	149	85.6	25	14.4	3.33	0.06
Yes	54	73.0	20	27.0		
Diabetes mellitus						
No	141	87.0	21	13.0	4.87	0.08
Type I	8	47.1	9	52.9		
Type II	54	78.3	15	21.7		
Asthma						
No	200	82.6	42	17.4	4.75	0.03^*^
Yes	3	50.0	3	50.0		
Renal disease						
No	199	83.3	40	16.7	1.18	0.27
Yes	4	44.4	5	55.6		
Hepatic disease						
No	202	82.1	44	17.9	0.08	0.78
Yes	1	50.0	1	50.0		
Neurological disorder						
No	199	81.9	44	18.1	0.24	0.62
Yes	4	80.0	1	20.0		
Malignancy						
No	202	81.8	45	18.2	0.27	0.60
Yes	1	100.0	0	0.0		
***Presenting COVID-19 symptoms***						
Fever						
No	75	93.8	5	6.3	4.49	0.03^*^
Yes	128	76.2	40	23.8		
Cough						
No	94	81.7	21	18.3	0.24	0.62
Yes	41	78.9	11	21.2		
Sore throat						
No	176	80.7	42	19.3	0.01	0.94
Yes	27	90.0	3	10.0		
Runny nose						
No	191	81.3	44	18.7	0.57	0.45
Yes	12	92.3	1	7.7		
Wheeze						
No	201	81.7	45	18.3	0.81	0.36
Yes	2	100.0	0	0.0		
Shortness of breath						
No	102	90.3	11	9.7	7.35	0.007^*^
Yes	101	74.8	34	25.2		
Fatigue						
No	195	81.6	44	18.4	0.03	0.85
Yes	8	88.9	1	11.1		
Muscle ache						
No	192	81.7	43	18.3	2.95	0.08
Yes	11	84.6	2	15.4		

^*^ Statistically significant at *p*<0.05.

Table 3 shows how survival was related to complete blood counts and coagulation factors. Low hemoglobin (*p*=0.01) and elevated D-Dimer (*p*=0.002) levels were significant predictors of death. All those with normal D-Dimer levels survived, but among those with elevated levels, the mortality rate was high.

**Table 3:** - Distribution of survival status in relation to baseline blood work-ups in the study individuals (N=248).

Parameter	Survivals		Non-survivors		Long rank test	*P*-value
	n	%	n	%		
***Hemoglobin***						
Normal	165	89.2	20	10.8	6.49	0.01^*^
Low	38	60.3	25	39.7		
***White blood cells***						
Normal	12	92.3	1	7.7	1.17	0.28
Leukocytosis	188	81.0	44	19.0		
***Ferritin***						
Normal	33	97.1	1	2.9	2.82	0.09
High	48	61.5	30	38.5		
***Platelets***						
Low	36	80.0	9	20.0	2.54	0.28
Normal	142	80.7	34	19.3		
High	21	91.3	2	8.7		
***aPTT***						
Low	13	92.9	1	7.1	3.83	0.14
Normal	102	77.3	30	22.7		
High	7	33.3	14	66.7		
***PTT***						
Normal	88	81.5	20	18.5	2.26	0.13
High	33	56.9	25	43.1		
***INR***						
Low	18	78.3	5	21.7	0.31	0.85
Normal	39	76.5	12	23.5		
High	65	69.9	28	30.1		
***D-Dimer***						
Normal	52	100.0	0	0.0	9.08	0.002^*^
High	151	77.0	45	23.0		
***Glucose***						
Normal	51	86.4	8	13.6	1.92	0.16
High	82	70.1	35	29.9		
***Hemoglobin A1C***						
Normal	9	100.0	0	0.0	0.62	0.43
High	36	83.7	7	16.3		

^*^ Statistically significant at *p*<0.05. PTT: partial thromboplastin time, aPTT: activated partial thromboplastin time, INR: international normalized ratio

A remarkable relationship was found between certain management protocols and death. The probability of survival beyond 20 days among those requiring invasive ventilation was approximately 60% lower compared to those not requiring ventilation. The group requiring tracheostomies had an 11-day median survival compared to a 22-day median survival for those not requiring tracheostomies. Those given chloroquine had about a 5% lower probability of survival beyond 20 days compared to those not given chloroquine; however, this was not statistically significant.

Table 4 shows that cardiovascular and respiratory complications were also related to survival time. ICU admission (*p*<0.001), pneumothorax (*p*=0.006), cardiac arrest (*p*<0.001), cardiac ischemia (*p*=0.01), cerebrovascular stroke (*p*<0.001), and bacteremia (*p*=0.01) were significant risk factors for death. Other complications related to the risk of death were acute renal failure and heart failure (*p*<0.001; Figures 1 E & F).

**Table 4:** - Distribution of survival status in relation to management protocol, complications, and treatment options in the study participants (N=248)

Parameter	Unit	Survivals		Non-survivors		Long rank test	*P*-value
		n	%	n	%		
***Management protocol***							
Intensive care unit admission	No	158	99.4	1	0.6	32.25	<0.001^*^
	Yes	45	50.6	44	49.4		
Non-invasive ventilation	No	163	81.1	38	18.9	3.17	0.07
	Yes	40	85.1	7	14.9		
Invasive ventilation	No	201	94.8	11	5.2	73.57	<0.001^*^
	Yes	2	5.6	34	94.4		
Inserted tracheostomy	No	202	86.7	31	13.3	16.11	<0.001^*^
	Yes	1	6.7	14	93.3		
Renal replacement therapy	No	200	83.0	41	17.0	0.17	0.67
	Yes	3	42.9	4	57.1		
***Complications***							
Pneumothorax	No	203	83.2	41	16.8	7.5	0.006^*^
	Yes	0	0.0	4	100.0		
Pleural effusion	No	197	82.8	41	17.2	1.13	0.28
	Yes	6	60.0	4	40.0		
Pulmonary embolism	No	199	84.0	38	16.0	1.89	0.18
	Yes	4	36.4	7	63.6		
Cardiac arrest	No	203	99.0	2	1.0	121.3	<0.001^*^
	Yes	0	0.0	43	100.0		
Cardiac ischemia	No	202	83.5	40	16.5	6.31	0.01^*^
	Yes	1	16.7	5	83.3		
Cardiac arrhythmia	No	202	83.1	41	16.9	2.16	0.14
	Yes	1	20.0	4	80.0		
Heart failure	No	203	83.5	40	16.5	67.48	<0.001^*^
	Yes	0	0.0	5	10.0		
Cerebrovascular stroke	No	203	83.5	40	16.5	12.09	<0.001^*^
	Yes	0	0.0	5	100.0		
Liver dysfunction	No	189	81.5	43	18.5	0.59	0.44
	Yes	14	87.5	2	12.5		
Acute renal failure	No	203	85.3	35	14.7	22.97	<0.001^*^
	Yes	0	0.0	10	100.0		
Bacteremia	No	202	83.5	40	16.5	5.93	0.01^*^
	Yes	1	16.7	5	83.3		
***Treatments***							
Ribavirin	No	182	81.6	41	18.4	0.34	0.55
	Yes	21	84.0	4	16.0		
Lopinavir-Ritonavir	No	159	83.3	32	16.8	0.15	0.69
	Yes	44	77.2	13	22.8		
Chloroquine	No	129	79.1	34	20.9	3.38	0.06
	Yes	198	81.5	45	18.5		
Corticosteroids	No	161	83.0	33	17.0	0.10	0.74
	Yes	42	77.8	12	22.2		
Heparin	No	123	89.1	15	10.9	2.64	0.10
	Yes	80	72.7	30	27.3		

^*^ Statistically significant at *p*<0.05.

The Cox proportional hazard model revealed that age, comorbid asthma, and acute renal failure and heart failure, as in-hospital complications, were independent predictors for death (Table 5). Those who were older (aged more than 50 years) were 2.26 times more likely to die than those who were younger after controlling for asthma, acute renal failure, heart failure, hemoglobin, and D-Dimer levels. Those with asthma had 3.87 times increase in hazard of death than those without after controlling for all other covariates. Also, those with acute renal failure as a complication of COVID-19 were 4.75 times more likely to die compared to those without renal failure after an adjustment for all other variables was made. Those with heart failure as a complication exhibited 50 times increase in the risk of death compared to those without this complication after controlling for all other variables. Patients with low hemoglobin levels were 1.82 times more likely to die compared to those with normal counts after controlling for other variables; however, this was not statistically significant (*p*=0.09). Subjects with elevated D-Dimer levels were 1.01 times more likely to die compared to those with normal D-Dimer levels after controlling for age, asthma, acute renal failure, anemia, and heart failure; this was marginally significant (*p*=0.05).

**Table 5:** - Cox proportional hazard model for the predictors of time-to-death among the hospitalized COVID-19 patients (N=248).

Variable	HR	95% CI	P-value	aHR	95% CI	*P*-value
Age – Ref: <50 years						
50+ years	2.05	1.07 – 3.94	0.03^*^	2.26	1.03 – 4.91	0.04^*^
Asthma – Ref: No						
Yes	3.43	1.04 – 11.29	0.04^*^	3.87	1.09 – 13.75	0.04^*^
Acute renal failure – Ref: No						
Yes	4.86	2.35 – 10.06	<0.001^*^	4.75	2.10 – 10.74	<0.001^*^
Hemoglobin – Ref: Normal						
Low	2.18	1.16 – 4.07	0.01^*^	1.82	0.89 – 3.69	0.09
Heart failure – Ref: No						
Yes	31.37	8.56 – 114.97	<0.001^*^	50.12	11.95 – 210.20	<0.001^*^
D-Dimer – Ref: Normal						
High	1.02	0.89 – 1.04	>0.05	1.01	0.99 – 1.03	0.05

^*^ Statistically significant at *p*<0.05. HR: hazard ratio, aHR: adjusted hazard ratio, CI: confidence interval, Ref: reference

## Discussion

A total of 248 patients were enrolled in this study. This study aimed to explain the in-hospital mortality among COVID-19 patients, using a survival analysis approach, in relation to pre-existing factors and factors existed at presentation or during hospitalization at the primary COVID-19 referral center in Jeddah. Among the 248 COVID-19 hospitalized patients, 203 (81.9%) were discharged to home and 45 (18.2%) died in the hospital before the end of the study period. Ccoronavirus disease 2019 infection can cause mild to severe symptoms and death.

Coronavirus disease 2019 non-survivors in our cohort were older than the survivors, which is consistent with previous research.^18,19^ Our cox-proportional hazard model showed that older COVID-19 patients were 2.26 more likely to die compared to younger patients after controlling for asthma, acute renal failure, anemia, heart failure, and D-Dimer levels. Older adults are more prone than younger ones to have comorbid conditions and severe COVID-19, requiring more medical attention. In several studies, older age has been the most important factor associated with hospitalization.^20,21^ Additionally, age is one of the variables that can affect the severity and the prognosis of the disease; it was shown that the elderly are more likely to be admitted to ICUs and have a higher mortality rate in compared to middle-aged patients. Lung anatomy changes, changes in physiological function including decreased lung reserve, decreased airway clearance, and decreased function of the protective lung barrier are age-related changes in the geriatric population.^22^ Elderly patients and those with underlying diseases have a higher risk of developing severe COVID-19 disease requiring intensive care; this can be explained by the chronic inflammatory response to the underlying disease causing unfavorable effects on immunomodulation and metabolic stress that potentially reduce the body’s ability to react to external agents, such as COVID-19 virus.^18-20^

Diabetes mellitus and hypertension have a somewhat positive association with COVID-19 and death. Previous research found a significant relationship between death and Covid-19 infection in patients who have a medical history of diabetes mellites.^23^ Multiple pathophysiological processes potentially support the link between DM and COVID-19 severity; however, much of what we know is based on SARS-COV infection rather than COVID-19. This association has been linked to a weakened innate immune system caused by chronic hyperglycemia, a pro-inflammatory state defined by an incorrect and excessive cytokine response and underlying pro-thrombotic hypercoagulability.^24,25^ The Centers for Disease Control and Prevention (CDC) uses COVID-NET to conduct population-based surveillance for laboratory-confirmed COVID-19-associated hospitalizations in the US. According to COVID-NET data, 89.3% of the admitted patients with COVID-19 have underlying comorbid condition. Obesity, hypertension, and diabetes mellitus were among the most frequently reported comorbidities.^26,27^ Systematic reviews and meta-analyses show that among all patients in the collected studies, hypertension was the prevalent underlying condition and its prevalence (56.8%) in the non-survival group was significantly higher than in survivors.^6,28^ A retrospective study of inpatients in Wuhan found that among different comorbidities, only hypertension has a positive association with the severity of COVID-19 after adjustment for age, gender, and smoking status.^12^

We found that underlying cardiac diseases had the largest effect on COVID-19 mortality. This is consistent with a previous study that showed those with CVD have double the risk of mortality.^24^ Another study found that 91% of COVID-19 fatalities involved patients with a past medical history of CVD.^29^ In non-survival groups, both myocardial injury and increased inflammation are more prevalent. Compared to COVID-19 survivors, higher troponin I levels are found to be present in non-survival groups. Other studies found that ~12% of hospitalized COVID-19 patients had underlying congenital heart defects/CVD, which were also correlated with a 3.8-fold increase in the odds of death.^6^ Chronic kidney disease appears to be one of the comorbidities that may affect mortality rates in patients admitted with COVID-19 infection; however, we did not find a significant association between mortality and history of chronic kidney disease. According to Cheng et al,^30^ 14.4% of those admitted to hospitals in China had high levels of creatinine. In China, in-hospital deaths constituted 33.7% of those with elevated baseline serum creatinine; this rate was substantially greater than of patients with normal baseline serum creatinine (13.2%).^30^

One of the comorbidities that increases the risk of mortality in patients admitted to the hospital with COVID-19 in Jeddah was asthma. In our cox-proportional model, patients with COVID-19 who had been diagnosed with asthma were 3.87 times more likely to die compared to their counterparts, after accounting for all other variables in the model. However, only 2.4% of our sample had asthma, which is lower than the asthma prevalence rate in the Kingdom of Saudi Arabia (3.5-27.5%).^31^ Santons et al^32^ found that patients who reported having asthma had a better survival rate compared to others, and they hypothesized that asthma may protect against severe COVID-19 infection. Another study carried out in Wuhan found a 0.9% asthma prevalence rate in COVID-19 patients, which is lower than the reported prevalence in the adult population of Wuhan (6.4%). The researchers hypothesized that the TH2 immune response in asthmatic patients may counteract the inflammation caused by SARS-CoV-2 infection.^12^ However, Further research is needed to confirm this hypothesis.

Regarding the symptoms that patients have at presentation, fever (67.7%) was the most frequent clinical symptom followed by shortness of breath (54.4%), cough (31.1%), and sore throat (12.1%). Fever and shortness of breath, amongst all clinical presentations, were related to mortality. Non-survivors presented more commonly with shortness of breath and fever than survivors. A group of researchers carried out a study and found that the critical group had more patients with fever and shortness of breath compared to other groups.^33^ Consistently, compared with survivors, non-survivors presented more commonly with higher respiratory rates, indicating that changes in vital signs should be monitored more closely.^18^ However, another study showed that clinical signs or symptoms, such as fever, cough, sore throat, diarrhea, or vomiting, were associated with a higher rate of survival and a lower chance of mortality.^32^

Research shows that a higher level of blood urea nitrogen, creatinine, albumin, bilirubin, alanine transaminase, and aspartate transaminase are more common in non-survival groups compared to survival groups. Mortality was also related to low platelet counts and elevated levels of D-dimer, indicating potential coagulopathy in COVID-19 patients. In addition, non-survival patients had a higher WBC count, lower lymphocyte, and cluster of differentiation subtypes (CD4+/CD8+) T-cell counts. Taken together, these results indicate that initial laboratory evaluation is important for risk stratification of COVID-19 patients.^6^ Our results indicate that high D-Dimer levels are somewhat associated with an increased risk of death. Non-survival groups also present with elevated levels of inflammatory markers, such as C-reactive protein (CRP), interleukin-6 (IL-6), lactate dehydrogenase (LDH), and erythrocyte sedimentation rate (ESR).^12,34,35^ These results will help healthcare workers to provide special care for patients with abnormal baseline laboratory results.

The management protocol is a good indicator of severity and mortality for COVID-19 patients. In our study, the proportion of patients admitted to the ICU was 35.9%, patients who needed invasive ventilation was 14.5%, and patients who needed an inserted tracheostomy was 6%. Intensive care unit admission, invasive ventilation, and inserted tracheostomy are indicators for a high risk of death in COVID-19 inpatients. This result is consistent with previous studies.^19,32^ Additionally, the treatments used during hospitalization may affect the mortality or the severity of the disease. However, in our study, the use of ribavirin, lopinavir–ritonavir, chloroquine, corticosteroids, or heparin did not provide any significant benefits in terms of mortality. Cao B, et al^36^ carried out a large randomized clinical trial to evaluate the efficacy and safety of oral lopinavir–ritonavir in admitted patients with COVID-19 infection, and their result showed no obvious benefits from using lopinavir–ritonavir compared to the standard-of-care.Consistently, Tian et al^33^ compared treatment options (antibiotics, anticoagulation, corticosteroid, and oxygen therapy) between critical and non critical patients, yet none of the treatments was proven to be effective.^33^

COVID-19 infection affects multiple organs in the body and can cause many complications in severe cases. Based on our findings, the most frequent complication was cardiac arrest (17.3%), followed by liver dysfunction (6.5%), pulmonary embolism (4.4%), pleural effusion (4.1%), and acute renal failure (4%). Furthermore, pneumothorax, cardiac arrest, cardiac ischemia, heart failure, cerebrovascular stroke, acute renal failure, and bacteremia had a significant association with death. However, only acute renal failure and heart failure, amongst all in-hospital complications, were independently associated with high mortality rate. Previous studies have confirmed many COVID-19-related complications such as cardiac arrest, liver and kidney dysfunction, and cardiomyopathy.^19,29,32^

### Study limitations

First, we collected our data from electronic medical records, which depend on the treating doctor, so some comorbidities may be underreported. Second, some blood tests were not completed in all patients. Third, not all of the baseline investigations were carried out on the date of admission, which may affect the results. Fourth, some patients were referred to our hospital from other centers, and previous treatments the patients may had received might alter our endpoints. We did not have an access to other centers’ laboratory investigations, and we considered the investigations performed at our hospital as the baseline investigations. Finally, the fact that there are some missing values is major limitation in the current study which is an inherent limitation for such charts review-based studies.

In conclusion, the association of death from COVID-19 with the presence of asthma and the old age is evident. In-hospital deterioration in heart and renal functions is linked to mortality. D-Dimer is a considerable parameter for predicting the mortality. This study did not show outcome differences in some treatment options. Healthcare workers should take special care of those with the aforementioned indicators to improve the survival. Further research on a larger scale and greater representation of hospitalized COVID-19 patients will be necessary.
